# Temporal Integration of Serum Proteomics, Metabolomics and MRI Tumor Volumetrics via Deep Learning Identifies Systemic Mediators of Glioblastoma Response to Chemoradiotherapy

**DOI:** 10.21203/rs.3.rs-9085743/v1

**Published:** 2026-03-18

**Authors:** Andra Krauze, Trinh Nguyen, Michael Sierk, Luke Jackson, Shreya Chappidi, Qingrong Chen, Chunhua Yan, Ying Hu, Stephanie Harmon, Erdal Tasci, Theresa Cooley, Mary Sproull, Megan Mackey, Daoud Meerzaman, Kevin Camphausen

**Affiliations:** National Cancer Institute, NIH; National Cancer Institute, NIH 9609 Medical Center Dr; National Cancer Institute, NIH 9609 Medical Center Dr; National Cancer Institute, NIH 9609 Medical Center Dr; National Cancer Institute, NIH; National Cancer Institute, NIH 9609 Medical Center Dr; National Cancer Institute, NIH 9609 Medical Center Dr; National Cancer Institute, NIH 9609 Medical Center Dr; National Cancer Institute, NIH 9609 Medical Center Dr; National Cancer Institute, NIH; National Cancer Institute, NIH; National Cancer Institute, NIH; National Cancer Institute, NIH; National Cancer Institute, NIH 9609 Medical Center Dr; National Cancer Institute, NIH

**Keywords:** glioma, radiation, proteomic, AI, MRI

## Abstract

**Background:**

Glioblastomas (GBM) are highly aggressive, treatment-resistant brain tumors lacking clinically actionable, noninvasive prognostic biomarkers. Tumor response after standard-of-care chemoradiation (CRT) is difficult to interpret on imaging, and post-CRT MRI changes have not been well linked to molecular features or potential biomarkers.

**Purpose:**

We evaluated differential proteomic and metabolomic expression in patient serum in relation to AI-segmented MRI volume changes after CRT to integrate clinical, molecular, and imaging data with patient outcomes.

**Materials and Methods:**

Fifty- five clinically annotated GBM patients provided serum samples pre- and post-CRT, analyzed using the SomaScan^®^ proteomic platform and SECIM metabolomic assay. Pathway signatures were derived from pre- vs. post-CRT differential expression. MRI scans underwent AI segmentation to quantify contrast-enhancing (CE), non-enhancing (NE), and edema volumes. We assessed correlations between early (immediately post-CRT) and late (six months post-CRT) imaging changes and molecular alterations. Integrated multiomic and imaging features were used for unsupervised clustering to identify survival-associated patient groups, followed by pathway re-identification.

**Results:**

AI-derived CE volumes decreased significantly during the early period, while edema increased significantly during the late period. CE changes were associated with metabolic pathways relevant to GBM biology, including epithelial–mesenchymal transition, in ammatory response, coagulation, and interferon-γ signaling. Clustering revealed two groups with distinct survival outcomes; CE alterations were significantly greater in the low-survival cluster (p = 0.02). Multiomic analysis (MOGSA) showed downregulation of key metabolic pathways in the low-survival group, including the citric acid cycle, Warburg effect, amino acid metabolism, oncogenic 2-hydroxyglutarate activity, and purine metabolism. Contributing metabolites included fumarate, succinate, citrate, and 2-hydroxyglutarate, while major proteomic contributors included MPC1, PDHB, DLAT, DLST, IDH3, SDHB, and FH.

**Conclusions:**

AI-derived MRI tumor-volume changes after CRT correspond to specific serum proteomic and metabolomic alterations, highlighting metabolic pathways linked to contrast-enhancing tissue dynamics in GBM.

## Introduction

Gliomas are highly treatment-resistant brain tumors. In glioblastoma (GBM) (WHO grade IV), standard of care requires surgical resection followed by concurrent (CRT) radiation therapy (RT) and temozolomide (TMZ), followed by adjuvant TMZ. Overall, the prognosis is poor with overall survival (OS) less than 30% at two years[1, 2], and there is currently no clinically applicable biomarker for GBM. Molecular classification in the form of MGMT methylation and IDH mutation, which, when present, now defines astrocytoma grade IV, as opposed to GBM, has proved difficult to connect to specific -omic alterations or imaging changes[3]. Previous attempts at integrating molecular and imaging classification have various limitations, including small datasets and a focus on a single modality. Most studies have limited clinical annotation[4], while studies in radiology have a paucity of both detailed clinical annotation and accompanying omic data[5–7]. Multichannel data integration studies that directly involve omic data in GBM are broadly comprised of studies that integrate a non-omic data type with an omic data type, e.g., radiology and genomic data [8–10] or histology and proteomic data [11], and studies that integrate several omic data streams without imaging data. Several tumor tissue-based studies have integrated various multiomics data from GBM tumor tissue: genomic and proteomic[12], genomic, transcriptomic, and proteomic[13], genomic, proteomic, and metabolomic [14], and radio-pathology and proteogenomic [15]. To date, multiomics studies have linked proteogenomic characterisation to survival[12], defined immune subtypes[14] and neuronal transition[13], and characterised tumors based on spatial proteomics[11]. The most critical limitations for genomic studies center on the inability to fully account for posttranslational modifications and epigenetic changes[16]. In addition, in brain tumors, the most practical barriers relate to the need to use tumor tissue for analysis. These barriers lead to results that are difficult to validate, perpetuate a limited biological understanding of tumor resistance, and undermine the ability to identify actionable biomarkers[3]. Noninvasive biospecimen analysis avoids both barriers, as it captures a downstream signal resulting from the integration of complex signaling pathways and can be easily measured over time, allowing mapping to the tumor state. Blood-based proteomic analysis has been performed in a variety of cancers, though integration with metabolomic data is rare[17]. In the clinic, tumor appearance on radiographic images is the most common method for assessing tumor recurrence, and imaging is the primary source of data used in standard-of-care disease management (aside from routine lab work). While plentiful, imaging data present multiple barriers to multimodal computational analysis. It is frequently housed in siloed data repositories, requires significant computational expertise to preprocess and extract clinically relevant information, and is typically difficult to link to the natural history of the disease. As a result, there are no triple-modality (proteome, metabolome, clinical imaging) studies in cancer, as studies that integrate imaging utilise histopathology images rather than clinical radiologic imaging. Hence, no studies have combined clinical and imaging data with serum proteomic and metabolomic data in GBM. The present study uniquely integrates serum proteomic, metabolomic, and MRI brain imaging alterations in patients with GBM to uncover potential proteomic and metabolomic biomarkers and critical pathways associated with survival outcomes.

## Materials and methods

### Patients, proteomic and metabolomic assays

Fifty-five patients with pathology-proven GBM diagnosed between 2005 and 2013, who enrolled on NCI NIH IRB-approved protocols and were treated with CRT, with available pre- and post-CRT serum samples and MRI imaging pre- and post-CRT available for AI volume segmentation, were included in the analysis (Supplemental Table 1). Mean age was 54 ( range 29–79). Twelve patients also received concurrent valproic acid (VPA) in addition to concurrent temozolomide and radiotherapy (RT) on protocol (the effect of VPA was analyzed and reported on separately in [18]).

**Figure 1:**
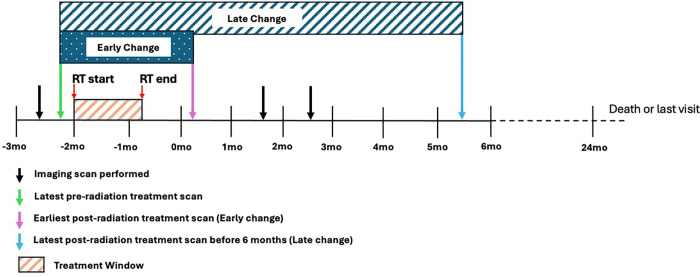
Designation of early and late volumetric changes in relationship to MRI brain scan acquisition during the natural history of the disease. The latest scan prior to the start of CRT serves as the baseline. In contrast, the earliest scan following completion of CRT serves as the post-timepoint for the Early Change (dark blue bar), and the last available scan prior to 6 months after the end of CRT serves as the post-timepoint for the Late Change (light blue bar).

Blood biospecimens were obtained before and after CRT. Serum samples were screened using the multiplexed, aptamer-based approach (SomaScan^®^ assay) in the SomaLogic^®^ research facility with sample specifications as per [19] using approximately 150 μL of serum[20, 21]. The relative concentrations of 7596 protein targets were measured, with 6405 unique proteins employed in the analysis for changes in expression. Clinical data were obtained or derived from the electronic health record. Metabolomic analysis was performed by the Southeast Center for Integrated Metabolomics (SECIM) in conjunction with the Department of Pathology, Immunology, and Laboratory Medicine at the University of Florida, Gainesville, FL, USA. It comprised 6015 compounds, of which 225 were annotated at level 1, indicating high confidence in the biological annotations based on peaks identified in the SECIM database. MRI scans of the brain, performed before tumor resection and after CRT, were selected for inclusion based on their timing relative to CRT administration. AI-derived CE, NE, and edema volumes were obtained as described in [22]. Early and late change volumetric differences in relationship to the treatment window with CRT were calculated as shown in Fig. 1. The clinical and proteomic dataset query and storage operations were provided by NIDAP[23], and data analysis was performed on Biowulf (https://hpc.nih.gov).

### Statistical Analysis

#### Proteomic pathway signatures and protein-protein interaction

We performed a paired t-test using post- vs. pre-CRT SomaScan^™^ RFU (Relative Fluorescent Units) values and calculated false discovery rate (FDR) values using the Benjamini-Hochberg method. Significantly upregulated (Log2FC > = 0.2, FDR < 0.05) and down-regulated (Log2FC <= −0.2, FDR < 0.05) proteins were fed to the R package OmicPath[24] for gene set analysis (GSA) against the HALLMARK, KEGG, and Reactome databases[25, 26], and protein-protein interaction network analysis. Pathways were considered significant if the FDR for the Normalized Enrichment Score was < 0.25.

#### Metabolomic pathway signatures

We performed a paired t-test using post- vs. pre-CRT compound measurements (moles/L) post data transformation by SECIM and calculated false discovery rate (FDR) values using the Benjamini-Hochberg method. Significantly upregulated (Log2FC > = 0.2, FDR < 0.25) and down-regulated (Log2FC <= −0.2, FDR < 0.25) compounds were entered into MetaboAnalyst 6.0 [27] through its web-based application[28] with default parameter settings to search for any significant metabolome pathways with FDR < 0.25.

#### Correlations of early change and late change of AI-Quantified Tumor Volumes and proteomics and metabolomics Profiles

We calculated changes in tumor (contrast-enhanced or non-contrast-enhanced) and edema volumes after CRT, either early (using the earliest scan after completion of CRT) or late (using the latest scan after completion of CRT prior to 6 months) (Fig. 1). We then identified significant proteins and compounds correlated with either the early change or late change using the cor.test function in R. Significantly positive correlated (correlation values > 0.3, p < 0.01) and negative correlated (correlation values <−0.3, p < 0.01) proteins were fed into R package OmicPath[24] for GSA against the HALLMARK, KEGG and Reactome databases [25, 26]. Pathways were considered significant if the FDR of the gene set was <0.05.

#### Multiple omics data integrative clustering

To identify subgroups for post-pre differences, we integrated proteomics and metabolomics expression data with early change tumor volumes. We performed the multiple factorial analysis (MFA)[29], following ConsensusClusterPlus with 4 PCs as distance, and with these parameter settings: maxK = 6, reps = 10000, pItem = 0.8, clusterAlg=“hc”, finalLinkage=“ward.D2”, distance=“pearson”, and defaults for all other parameters.

#### Multiple omics gene set analysis (MOGSA)

We used the post-pre differences from the proteomics and metabolomics data as input to MOGSA, an R software package for multimodal single-sample gene set analysis [30]. Specifically, we used the MOGSA function to identify metabolite-protein pathway gene set scores (GSS) with the following parameter settings: nf = 4 (4 PCs selected), proc.row=”center_ssq1”, w.data= “lambda1”, and statis=FALSE. To choose representative molecular pathways from the resulting subgroups, we first selected the pathways with GSS FDR (false discovery rate) values smaller than 0.25 in 50% of all samples. We also applied a Wilcoxon test and selected pathways with an FDR < 0.01. Finally, we plotted GSS z-scores in a heatmap to show the patterns of pathway enrichment from both data types.

#### Pathway database

To study metabolite-protein pathway gene sets, we downloaded the CSV le containing all metabolite-pathway links (pathbank_all_metabolites.csv ) and the file containing all protein pathway links (pathbank_primary_proteins.csv) from PathBank[31]. These files were created on Oct 18, 2019. After that, for each common gene set, we selected the “KEGG ID” from the metabolite file and the “Gene Name” from the protein file if it is from the “Human” species.

## Results

### Tumor volume changes, differential protein and metabolite expression, and pathway analysis before and after CRT

Differential protein expression after CRT was observed with 282 proteins significantly altered with FDR < 0.05(Supplemental Fig. 1, Supplemental le 1). Differential metabolite expression was also observed; however, only 12 compounds were altered with FDRs ranging from 0.08 to 0.24 (Supplemental Fig. 2A, Supplemental le 2). The differentially expressed proteins are associated with several cancer hallmark pathways, including Il6_JAK_STAT3 signaling (FDR = 0.002), oxidative phosphorylation (FDR = 0.004), adipogenesis (FDR = 0.024), while glycolysis, xenobiotic metabolism, epithelial-mesenchymal transition, and MYC targets V2 all had FDR > 0.05. The differentially expressed metabolites were associated with purine metabolism (FDR 0.017). (Pyrimidine, caffeine, and porphyrin metabolism were also identified, but with FDR values > 0.25. (Supplemental Fig. 2B, Supplemental file 2)

**Figure 2:**
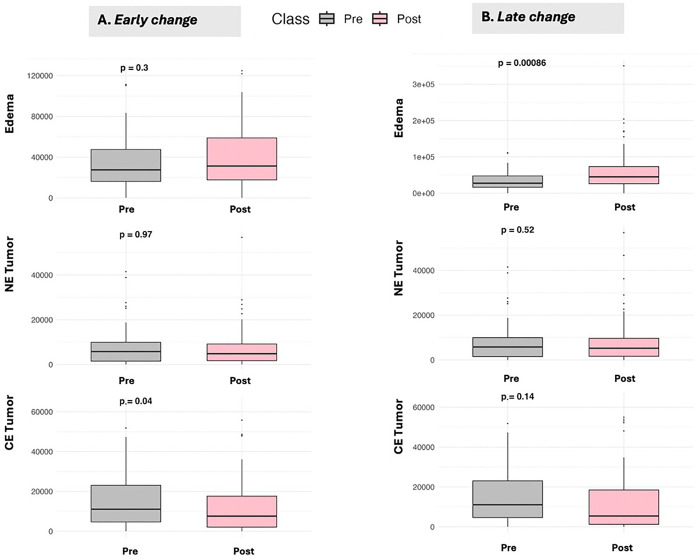
AI determined volume changes compared pre vs. post CRT for the early period (**A.** ) and late period (**B.** ). P values were derived from a paired t-test. NE Tumor = Non-contrast-enhancing tumor volume. CE Tumor = Contrast-enhancing tumor volume.

We compared changes in CE tumor, NE tumor, and edema volumes in the early (Fig. 2A) and late time periods (Fig. 2B). In the early change, only the CE tumor was statistically significant (decreased from pre to post). In the late change, only edema was statistically significant (increased from pre to post) (Fig. 2), although the direction of change was the same in the non-significant time intervals.

Several differentially expressed proteins were significantly correlated to volume changes in both the early and late intervals. The largest number of proteins were significantly correlated with CE volume in both the early (112) and late (83) intervals (with 65 proteins shared between the two), followed by NE (65 and 46, respectively) and edema (12 and 39, respectively) (Supplemental Table 2). To assess the significance of the number of proteins found in both early and late periods we performed a hypergeometric test, yielding p-values of 2.57e-07, 0.71, and 1 for CE, NE, and edema, respectively. Only one protein was associated with all three volume changes in both the early (PENK) and late (ApoM) intervals (Supplemental Table 3, Supplemental File 3). We performed Kaplan-Meier survival analysis based on *ApoM* and *PENK* expression to determine whether these proteins were potential biomarkers. Lower levels of *ApoM* were associated with a statistically significant improvement in both OS and PFS. *PENK* was not statistically significantly associated with either progression or survival (Supplemental Fig. 3). Pathway analysis of proteins significantly correlated with changes in tumor volume revealed several relevant pathways (Supplemental File 3). Pathways associated with CE tumor volume in both the early and late intervals include epithelial-mesenchymal transition, inflammatory response, coagulation, and interferon gamma response. IL2_STAT5 signaling, hypoxia, and myogenesis are associated with CE volume changes in the late interval. Epithelial-mesenchymal transition and hypoxia are also associated with edema changes in the early time interval. UV_response_UP is associated with both NE and edema in the late interval (Table 1). Several pathways were associated with 2 of the three volumes, but none were shared among all 3 (Supplemental File 3).

### Clustering of patients based on proteomic, metabolomic, and imaging alterations

**Figure 3:**
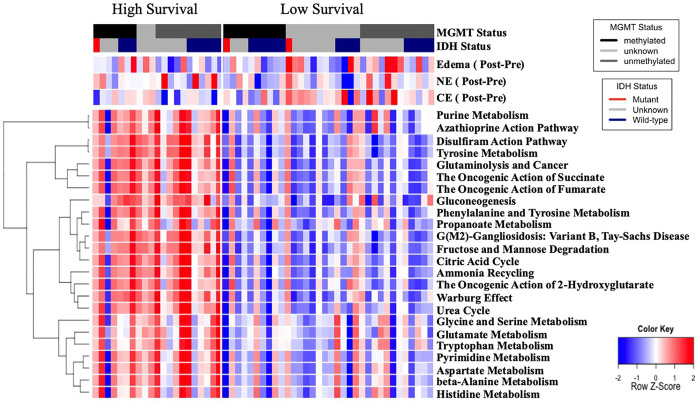
Heat map of differential expression of select pathways between high- and low-survival groups of patients. Z-scores are derived from the combination of proteomic and metabolomic data. NE = Non-contrast-enhancing disease; CE = Contrast-enhancing disease.

**Figure 4:**
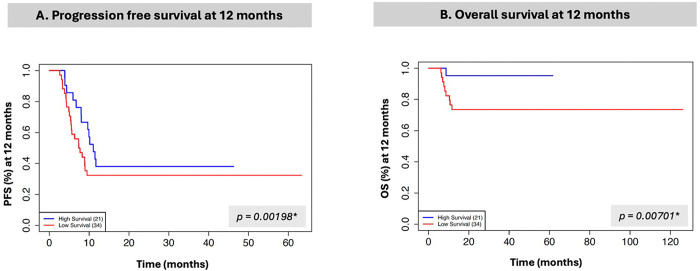
Kaplan-Meier curves of two patient subgroups. A. Progression-free survival at 12 months. B. Overall survival at 12 months.

Since there was substantial correlation between the volumes in the early and late time intervals (Supplemental Table 2), we used the early period to perform unsupervised clustering of patients based on the proteomic, metabolomic, and imaging features. This produced two clusters of patients (Fig. 3, Supplemental Fig. 4), which have statistically significant differences in both progression and overall survival (Fig. 4). We then examined which features were correlated with these survival groups (Supplemental Table 4). Of the three segmented tumor volumes, only CE showed a statistically significant difference between the low and high survival groups, with CE significantly lower in the high survival group (p = 0.02, FDR < 0.01).

Several metabolism-related pathways were identified as statistically significantly different based on either metabolite or proteomic data (Fig. 3). Purine metabolism, the citric acid cycle, 2-hydroxyglutarate-related2-hydroxyglutarate-related pathways, the Warburg effect, and multiple amino acid metabolism pathways were downregulated in the low-survival cohort (Fig. 3).

**Figure 5:**
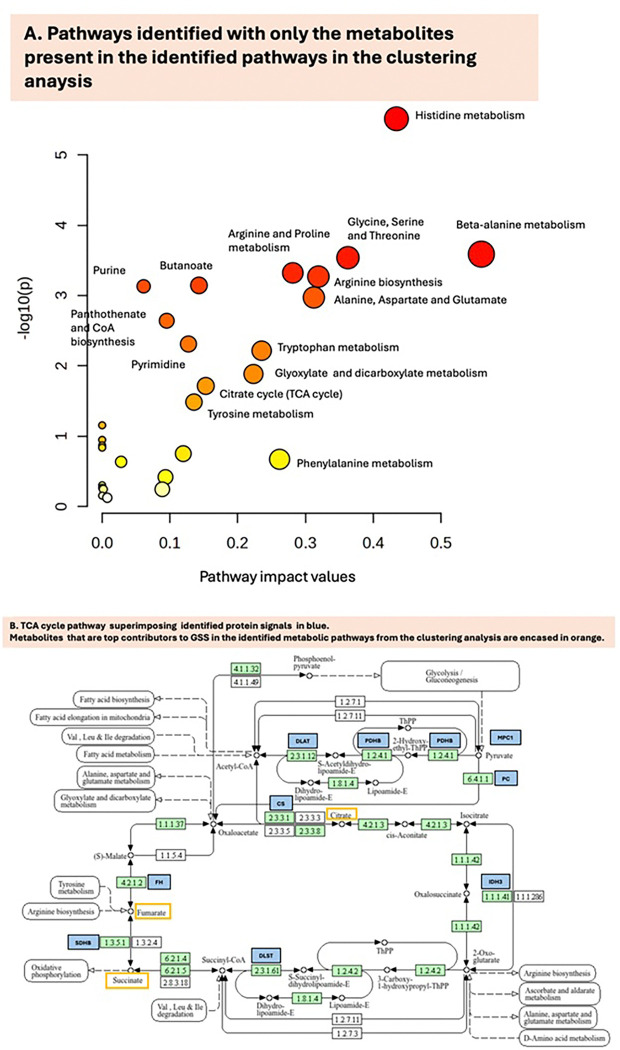
**A.** Pathways identified with the metabolites present in the clustering analysis (visualization in MetaboAnalyst[27]). Colors varying from yellow to red represent data with different levels of significance, from light yellow (higher p-values) to red (lower p-values). **B.** TCA cycle pathway superimposing identified protein signals in blue. Metabolites that are top contributors to GSS in the identified metabolic pathways from the clustering analysis are encased in orange.

Several proteins and metabolites emerged as essential contributors to the MOGSA gene-set scores, though metabolites consistently contributed more to the scores (Supplemental Fig. 5). Of the 25 pathways identified, 22 pathways had metabolites as the top contributors to the GSS. These included fumarate, citric acid, succinate, and several amino acids or amino acid precursors (Supplemental le 4). Several proteins and metabolites, including the Mitochondrial pyruvate carrier 1 (*MPC1*), were part of multiple signaling pathways that were downregulated in the low-survival group, including the 2hydroxyglutarate oncogenic pathway, citric acid cycle, and Warburg effect pathways (Fig. 5).

## Discussion

Despite the rapid growth of diverse data sources, including electronic health records (EHRs), imaging data, and various types of high-throughput molecular data, these sources individually have not yielded clear, consistent biomarkers for glioblastoma. While there have been efforts to integrate different data types into multimodal analyses that employ tissue-level and public data to enhance tumor subtyping or predict survival [12, 15, 32–35], there is still a need to explore different strategies for data integration to derive prognostic signatures, particularly using noninvasively acquired biospecimens. Here, we present a novel multimodal analysis demonstrating that such integration can yield new insights into the mechanisms underlying GBM prognosis. To our knowledge, this is the first study to combine clinical data with serum-derived proteomic and metabolomic data and to leverage pre- and post-treatment radiographic images using artificial intelligence-based segmentation and tumor volume calculation.

While classification of tumor progression in the clinic relies on radiographic images and clinical information, there is no standardized method for quantifying changes in tumor volume or other relevant characteristics[36]. AI algorithms have been shown to provide a variety of insights into imaging data and could be used to quantify clinically pertinent changes of GBM tumors[37, 38]. If images were collected at standardized timepoints throughout the treatment and observation protocols, this would further enhance our ability to glean information from patient cohorts. While the imaging data we use here are not entirely standardized in terms of collection timepoints, they suffice to demonstrate that images collected at appropriate timepoints can be correlated with molecular data.

Given that several factors could potentially affect changes in imaging-derived features, including variability in the timing of scans, the possibility of pseudo-progression after CRT, and the fact that treatment-related changes may persist long-term, we elected to examine changes at two different time points: early change using the earliest scan after CRT and late change using the newest scan within 6 months of CRT. In the clinic, alterations in brain MRIs after completion of CRT can be challenging to interpret as they may indicate tumor progression or treatment effect. Generally, the ability to assign actual progression to imaging changes requires either a tissue sample proving the presence of an active tumor or rapid imaging follow-up to determine if the alteration continues to progress, remains the same, or decreases. Contrast enhancement can increase or decrease post-CRT, whereas edema, as exempli ed by T2/FLAIR signal abnormality, often increases in the long term as treatment-related changes become established[39]. Consistent with this, we found that CE tumor volumes decreased during the early period (within 3 months of CRT completion) and edema volumes increased during the late period.

We identified significant differential expression of multiple proteins and metabolites in patient serum samples, and several of the DE proteins were associated with AI-segmented volumes in the early and late change analysis. *PENK* (proenkephalin) was shared amongst all volume changes in the early period but was not associated with survival in the study population. *PENK* has no known connection to glioma or GBM, but has been identified as a tumor suppressor gene and a potential biomarker in other cancers[40, 41]. There is also evidence that PENK is released by cultured astrocytes in a cell cycle-dependent fashion[42].

*ApoM* (Apolipoprotein M) was identified as correlated with all late-volume changes. *ApoM* is one of three carrier proteins for *S1P* (the other two are albumin and apolipoprotein A4), and S1P has been shown to induce Akt activation when bound to *ApoM* and is a critical component of sphingolipid metabolism, with wide-ranging signaling effects including proliferation, migration, and stem cell behavior [43, 44]. In the current study, patients with a decrease in serum *ApoM* post CRT exhibited superior PFS and OS, consistent with its biological activity.

The identified proteins showed significant correlation with the AI-segmented volumes and represented multiple relevant pathways. Epithelial-mesenchymal transition, inflammatory response, coagulation, interferon gamma response, and IL2_STAT5 signaling were associated with CE volume changes in both the early and late periods. The hypoxia pathway was related to early change edema, while UV_response_UP was associated with edema and NE volume changes in the late period. The identification of these pathways with biological significance in GBM, in conjunction with imaging alterations, suggests that it may be possible to detect serum biomarkers that reflect both tumor biology and imaging changes[45].

The two patient groups with differential survival identified via unsupervised clustering differ in the up-(high survival) or down- (low survival) regulation of critical metabolic pathways, including the citric acid cycle, the Warburg effect, amino acid metabolism, and the oncogenic action of 2-hydroxyglutarate. The decrease in these major metabolic pathways in the low-survival group, particularly in pathways where a decrease would intuitively be associated with improved survival, such as the Warburg effect, poses a complex challenge for interpreting the signal captured in serum. The Warburg effect describes the fermentation of glucose to lactate even in the presence of oxygen, termed aerobic glycolysis, with the goal of rapid energy production to power cell proliferation and, potentially, enhanced biosynthesis. The Warburg effect, however, is heterogeneous in cancer and remains poorly understood, with several theories described in detail by Liberti et al[46]. The observed pathway assignment for the Warburg effect, the citric acid cycle, and the action of 2-hydroxyglutarate is, in the present study, strongly attributable to 10 shared proteins (CS, DLAT, DLD, DLST, FH, IDH3G, MPC1, PC, PDHB, SDHB) and three shared metabolites (citric, fumaric, and succinic acid) that are heavily linked to each other. Noteworthy here is the observation that signal transduction as a consequence of the Warburg defect may differ in patients with glioma phenotypes, and that all of the above proteins are actually mitochondrial matrix proteins that do not typically circulate freely in serum. However, there have been studies that have investigated mitochondrial involvement in the progression of GBM and it has been shown that mitochondira are shared between cells within the tumor microenvironment and induce metabolic reprograming away from glycolysis and towards protein metabolism and pyrimidine metabolism, increasing agressivness and resistance, in line with our results[47, 48].

When employing Somascan, the detection limit may be lower, and these signals may also originate from microvesicles or exosomes. The observed directionality may be attributable to mitochondrial dysfunction in the lower-survival group. While fewer metabolites were differentially expressed, metabolites were the top contributors to the integrated analysis in the majority of the pathways. MOGSA integrates proteomic and metabolomic data, allowing it to contribute equally to the analysis; however, in the present study, annotated metabolites are fewer than the number of proteins available, which gives metabolites a much higher per-feature leverage. Nonetheless, the metabolic data signals identified in the present study were centered on citric, fumaric, and succinic acid, which are core TCA intermediates in classical metabolic circuits. If they exhibit coordinated variation that dominates, this can lead to pathway scores with those components being metabolite-driven, rather than proteins that function as catalysts, which show more noisy signals and possibly weaker variation overall (Fig. 5). In addition, proteins may exhibit greater redundancy, with several proteins implicated in driving a reaction.

In contrast, the metabolic intermediate steps are typically far fewer and map more directly to metabolic circuits. In this context, fumarate, succinate, and 2-hydroxyglutarate are also well-established as signaling molecules in cancer, with documented roles in GBM and glioma [49] [50]. In addition, proteomic signals (e.g., *MPC1*[51–53], *PDHB*[54], *DLAT*[55], *DLST*, *IDH3*[56], *SDHB*, and *FH*) reinforce the connection between metabolites and proteins by mediating the ow of carbon and energy. This signaling pattern is directly linked to proliferation in cancer, specifically in GBM, and is critical to metabolic reprogramming [49]. The oncogenic role of 2-hydroxyglutarate and glutamate metabolism found here is consistent with previous findings in glioma, including a similar relationship between metabolic pro les and survival [57, 58].

Among the top identified proteins, Mitochondrial pyruvate carrier 1 (*MPC1*) was found to be involved in several signaling pathways downregulated in the low-survival group, including the 2-hydroxyglutarate oncogenic pathway, the citric acid cycle, and the Warburg effect. *MPC1* encodes a protein that enables the transport of pyruvate into the mitochondria, a known mechanism for redirecting the energy currency in gliomas[53]. Data support the hypothesis that decreased *MPC1* may result in post-treatment glioma tumor growth by enabling metabolic reprogramming and driving proliferation. It has also been associated with the proneural subgroup of GBM, MGMT expression levels, and a diminished response to temozolomide[52]. These findings connect *MPC1* to both MGMT and IDH status in glioma and to metabolic mechanistic patterns, including carbon ow, energy currency, and the Warburg effect. Although not yet associated with prognosis in GBM, *MPC1* is a potential biomarker in other cancers, having been identified as both a mediator of metabolic processes via mTOR activation and a promoter of stem cell-like properties, with *MPC1* knockdowns and silencing resulting in larger tumors[51].

### Study limitations

The limitations of the study include the small sample size, given the desire to integrate clinical, omic, and imaging data. Imaging data before CRT, in particular, is critical; however, patients may undergo these initial scans at different institutions than the one where they are ultimately treated with CRT, making it difficult to standardise images for inclusion in AI segmentation due to differences in imaging protocols and file export. MGMT and IDH status were unknown in a large proportion of patients, reflecting a period of diagnosis (2005–2013) when molecular classification was not available for many patients. Only 5% of patients in the cohort were known IDH-mutated, and these patients today would be considered astrocytoma grade IV. The timing of serum biospecimen acquisition and MRI brain imaging rarely coincided with pre-op, post-CRT, or post-RT, and samples were not available for analysis beyond the immediate post-CRT time, limiting comparisons of proteomic changes to image changes that occurred several months into the study. The metabolomic data were limited to only a small proportion of compounds (< 5%) that were biologically annotated with level 1 confidence and a KEGG ID. We also do not exclude the possibility that additional markers contributing to the same pathways identified here, but not measured or annotated in the current study, could alter the observed results, if available. As annotating metabolomic datasets is becoming more expansive, conclusions may be augmented by evolving large-scale proteomic and metabolomic datasets.

## Conclusion

The serum proteome and metabolome exhibit biologically relevant connections to AI-derived volume evolution post-CRT in GBM. This can provide an avenue for identifying biomarkers from noninvasively collected biospecimens that are linked to imaging changes, which are the primary modes of tumor visualisation and clinical follow-up. Data integration enables the identification of metabolic pathways comprising both metabolic intermediates and proteomic enzymatic steps that operate together to produce observable changes in association with the outcome. Further validation can help drive the development of interventions to overcome tumor resistance by leveraging noninvasively derived biomarkers.

## Supplementary Material

This is a list of supplementary fi les associated with this preprint. Click to download.

• Supplementaryfile1.xlsx

• Supplementaryfile3.xlsx

• Supplementaryfile2.xlsx

• Supplementaryfile4.xlsx

• CGBBSupplementalFIguresandTables.pptx

• Supplementalmaterial.docx

## Figures and Tables

**Table 1:** Signaling pathways associated with differentially expressed proteins that are correlated with AI-segmented tumor volumes using an FDR cutoff of 0.05.

Pathway	Protein(s)	FDR
Contrast Enhancement (CE) Tumor Volume		
Early Change		
HALLMARK_EPITHELIAL_MESENCHYMAL_TRANSITION	SERPINE1, SPP1, MMP3, FBLN1, SFRP4, CXCL12	0.021
HALLMARK_INFLAMMATORY_RESPONSE	CCL2, CCL5, TNFSF10,TNFAIP6, SERPINE1,SELENOS	0.021
HALLMARK_INTERFERON_GAMMA_RESPONSE	TNFSF10, TNFAIP6, CCL5, CCL2, VAMP8	0.048
HALLMARK_COAGULATION	SERPINE1, MMP3, GNG12, RABIF	0.048
Late Change		
HALLMARK_EPITHELIAL_MESENCHYMAL_TRANSITION	MMP3, FBLN1, SFRP4, CTHRC1, MMP1	0.014
HALLMARK_INLAMMATORY_RESPONSE	CCL2, TNFSF10, TNFAIP6, GPC3, SELENOS	0.014
HALLMARK_COAGULATION	MMP1, MMP3, GNG12, RABIF	0.014
HALLMARK_IL2_STAT5_SIGNALING	DHRS3, NDRG1, PENK, TNFSF10	0.028
HALLMARK_HYPOXIA	NDRG1, GPC3, GAA, PAM	0.028
HALLMARK_MYOGENESIS	TPM3, GAA, AKT2, EFS	0.0278
HALLMARK_INTERFERON_GAMMA_RESPONSE	TNFSF10, TNFAIP6, CCL2, VAMP8	0.028
Non-Enhancing (NE) Tumor Volume		
Early Change		
HALLMARK_COAGULATION	C1R, F13B, GP1BA, HTRA1	0.028
Late Change		
HALLMARK_UV_RESPONSE_DN	COL3A1, TGFBR2	0.005
HALLMARK_UV_RESPONSE_UP	GLS, APOM	0.005
HALLMARK_MYOGENESIS	COL3A1, EPHB3	0.006
Edema Volume		
Early Change		
HALLMARK_HYPOXIA	PPT1, ZW10	0.00088
Late Change		
HALLMARK_PROTEIN_SECRETION	PPT1, ZW10	0.012
HALLMARK_UV_RESPONSE_UP	APOM, PPT1	0.018
HALLMARK_APOPTOSIS	TNFRSF12A, PPT1	0.018
HALLMARK_EPITHELIAL_MESENCHYMAL_TRANSITION	SPARC, TNFRSF12A	0.019
HALLMARK_XENOBIOTIC_METABOLISM	CYB5A, CBR1	0.019

A few metabolites showed significant correlations with volume changes (Supplemental File 3). In the early interval, gluconate (edema), quinoline (NE), and cortisol (CE) were significantly correlated, while in the late interval, hypoxanthine (edema), hydroxyphenylacetic acid, and quinoline (NE) were significantly correlated. Quinoline was negatively correlated with NE in both the early and late analyses.

## Abbreviations

CRT: Concurrent chemoradiation
DE: Differentially Expressed
EMT: Epithelial–Mesenchymal Transition
FDR: False Discovery Rate
GBM: Glioblastoma
GO: Gene Ontology
GSA: Gene set analysis
GSS: Gene set score
GTR: Gross Total Resection
KEGG: Kyoto Encyclopedia of Genes and Genomes
KOBAS: KEGG Orthology–Based Annotation System
MFA: Multiple Factor Analysis
MGMT: O6–Methylguanine–DNA Methyltransferase
MSigDB: The Molecular Signatures Database
MOGSA: Multiple omics gene set analysis
NES: Normalized Enrichment Score
OS: Overall Survival
PFS: Progression Free Survival
RANO: Response Assessment in Neuro–Oncology
RFU: Relative Fluorescent Units
RPA: Recursive Partitioning Analysis
RT: Radiation Therapy
RTOG: Radiation Therapy Oncology Group
SECIM: Southeast Center for Integrated Metabolomics
ssGSEA: Single–sample Gene Set Enrichment Analysis
STR: Subtotal Resection
TMZ: Temozolomide
UPR: Unfolded Protein Response
VPA: Valproic Acid
WHO: World Health Organisation

## References

[1] Senders J.T., , An Online Calculator for the Prediction of Survival in Glioblastoma Patients Using Classical Statistics and Machine Learning. Neurosurgery, 2020. 86(2): p. E184–E192. 31586211 10.1093/neuros/nyz403 PMC7061165

[2] Zeng J D.K., Krauze AV. Patterns of failure and development of a novel prognostic scoring system in elderly patients with glioblastoma – follow up on 10 year analysis of the BC cancer agency population. in Society of Neuro-Oncology 2019. 2019. Neuro-Oncology.

[3] Linhares P., , Glioblastoma: Is There Any Blood Biomarker with True Clinical Relevance? Int J Mol Sci, 2020. 21(16). 10.3390/ijms21165809 PMC7461098 32823572

[4] Fitt B., , Analytic approaches to clinical validation of results from preclinical models of glioblastoma: A systematic review. PLoS One, 2022. 17(3): p. e0264740. 10.1371/journal.pone.0264740 PMC8887747 35231064

[5] Fan H., , Artificial intelligence-based MRI radiomics and radiogenomics in glioma. Cancer Imaging, 2024. 24(1): p. 36. 38486342 10.1186/s40644-024-00682-y PMC10938723

[6] Bakas S., , The University of Pennsylvania glioblastoma (UPenn-GBM) cohort: advanced MRI, clinical, genomics, & radiomics. Sci Data, 2022. 9(1): p. 453. 35906241 10.1038/s41597-022-01560-7 PMC9338035

[7] Jia X., , A Multiparametric MRI-Based Radiomics Nomogram for Preoperative Prediction of Survival Stratification in Glioblastoma Patients With Standard Treatment. Front Oncol, 2022. 12: p. 758622. 10.3389/fonc.2022.758622 PMC8888684 35251957

[8] Fathi Kazerooni A., , The radiogenomic and spatiogenomic landscapes of glioblastoma and their relationship to oncogenic drivers. Communications Medicine, 2025. 5(1): p. 55. 40025245 10.1038/s43856-025-00767-0 PMC11873127

[9] Guo J., , Integrating imaging and genomic data for the discovery of distinct glioblastoma subtypes: a joint learning approach. Scientific Reports, 2024. 14(1): p. 4922. 38418494 10.1038/s41598-024-55072-y PMC10902376

[10] Qian X., , Radiogenomics-Based Risk Prediction of Glioblastoma Multiforme with Clinical Relevance. Genes (Basel), 2024. 15(6). 10.3390/genes15060718 PMC11202835 38927654

[11] Duhamel M., , Spatial analysis of the glioblastoma proteome reveals specific molecular signatures and markers of survival. Nat Commun, 2022. 13(1): p. 6665. 36333286 10.1038/s41467-022-34208-6 PMC9636229

[12] Yanovich-Arad G., , Proteogenomics of glioblastoma associates molecular patterns with survival. Cell Rep, 2021. 34(9): p. 108787. 10.1016/j.celrep.2021.108787 33657365

[13] Kim K.H., , Integrated proteogenomic characterization of glioblastoma evolution. Cancer Cell, 2024. 42(3): p. 358–377.e8. 38215747 10.1016/j.ccell.2023.12.015 PMC10939876

[14] Wang L.B., , Proteogenomic and metabolomic characterization of human glioblastoma. Cancer Cell, 2021. 39(4): p. 509–528.e20. 33577785 10.1016/j.ccell.2021.01.006 PMC8044053

[15] Liu Z., , Multimodal fusion of radio-pathology and proteogenomics identify integrated glioma subtypes with prognostic and therapeutic opportunities. Nature Communications, 2025. 16(1): p. 3510. 10.1038/s41467-025-58675-9 PMC11994800 40222975

[16] Koo H. and Sa J.K., Proteogenomic Insights Into Glioblastoma Evolution: Neuronal Reprogramming and Therapeutic Vulnerabilities. Brain Tumor Res Treat, 2025. 13(3): p. 81–86. 40759475 10.14791/btrt.2025.0018 PMC12329232

[17] An R., , Integrative analysis of plasma metabolomics and proteomics reveals the metabolic landscape of breast cancer. Cancer & Metabolism, 2022. 10(1): p. 13. 10.1186/s40170-022-00289-6 PMC9382832 35978348

[18] Krauze A.V., , Revisiting Concurrent Radiation Therapy, Temozolomide, and the Histone Deacetylase Inhibitor Valproic Acid for Patients with Glioblastoma-Proteomic Alteration and Comparison Analysis with the Standard-of-Care Chemoirradiation. Biomolecules, 2023. 13(10). 10.3390/biom13101499 PMC10604983 37892181

[19] Krauze Av, S.M.N.T.C.Q.Y.C.H.Y.J.W.T.E.C.Z.T.S.M.M.M.S.U.M., Glioblastoma survival is associated with distinct proteomic alteration signatures post chemoirradiation in a large-scale proteomic panel. Frontiers in Oncology, 2023. 10.3389/fonc.2023.1127645 PMC10448824 37637066

[20] Gold L., , Advances in human proteomics at high scale with the SOMAscan proteomics platform. N Biotechnol, 2012. 29(5): p. 543–9. 22155539 10.1016/j.nbt.2011.11.016

[21] Tuerk C. and Gold L., Systematic evolution of ligands by exponential enrichment: RNA ligands to bacteriophage T4 DNA polymerase. Science, 1990. 249(4968): p. 505–10. 2200121 10.1126/science.2200121

[22] Belue M.J., , Diagnosing Progression in Glioblastoma-Tackling a Neuro-Oncology Problem Using Artificial-Intelligence-Derived Volumetric Change over Time on Magnetic Resonance Imaging to Examine Progression-Free Survival in Glioblastoma. Diagnostics (Basel), 2024. 14(13). 10.3390/diagnostics14131374 PMC11241823 39001264

[23] Palantir Foundry—The NIH Integrated Data Analysis Platform (NIDAP); NCI Center for Biomedical Informatics & Information Technology (CBIIT); software provided by Palantir Technologies Inc., https://www.palantir.com.

[24] CBIIT-CGBB. OmicPath: an R package for gene set analysis and pathway network analysis. 2025 [cited 2025 8/1/2025]; Available from: https://github.com/CBIIT-CGBB/OmicPath.

[25] Subramanian A., , Gene set enrichment analysis: a knowledge-based approach for interpreting genome-wide expression proles. Proc Natl Acad Sci U S A, 2005. 102(43): p. 15545–50. 16199517 10.1073/pnas.0506580102 PMC1239896

[26] Mootha V.K., , PGC-1alpha-responsive genes involved in oxidative phosphorylation are coordinately downregulated in human diabetes. Nat Genet, 2003. 34(3): p. 267–73. 12808457 10.1038/ng1180

[27] Pang Z., , MetaboAnalyst 6.0: towards a unified platform for metabolomics data processing, analysis and interpretation. Nucleic Acids Res, 2024. 52(W1): p. W398–w406. 38587201 10.1093/nar/gkae253 PMC11223798

[28] MetaboAnalyst. MetaboAnalyst PathUploadView. 2025 8/1/2025 [cited 2025 8/1/2025]; PathUploadView]. Available from: https://dev.metaboanalyst.ca/MetaboAnalyst/upload/PathUploadView.xhtml.

[29] Abdi A., Williams L., and Valentin D., Multiple factor analysis: Principal component analysis for multi-table and multi-block data sets. Wiley Interdisciplinary Reviews: Computational Statistics, 2013. 5: p. 149–179.

[30] Meng C., , MOGSA: Integrative Single Sample Gene-set Analysis of Multiple Omics Data. Mol Cell Proteomics, 2019. 18(8 suppl 1): p. S153–s168. 31243065 10.1074/mcp.TIR118.001251 PMC6692785

[31] PathBank. Metabolite Pathway Links and Protein Pathway Links. 2019 [cited 2019 10/18/2019]; Available from: https://www.pathbank.org/downloads.

[32] Chouleur T., , A strategy for multimodal integration of transcriptomics, proteomics, and radiomics data for the prediction of recurrence in patients with IDH-mutant gliomas. Int J Cancer, 2025. 157(3): p. 573–587. 40214613 10.1002/ijc.35441 PMC12141979

[33] Li J., , Integrative analysis of metabolism subtypes and identification of prognostic metabolism-related genes for glioblastoma. Biosci Rep, 2024. 44(3). 10.1042/BSR20231400 PMC10965397 38419527

[34] Syafruddin S.E., , Integration of RNA-Seq and proteomics data identifies glioblastoma multiforme surfaceome signature. BMC Cancer, 2021. 21(1): p. 850. 34301218 10.1186/s12885-021-08591-0 PMC8306276

[35] Molinaro A.M., , Association of Maximal Extent of Resection of Contrast-Enhanced and Non-Contrast-Enhanced Tumor With Survival Within Molecular Subgroups of Patients With Newly Diagnosed Glioblastoma. JAMA Oncol, 2020. 6(4): p. 495–503. 32027343 10.1001/jamaoncol.2019.6143 PMC7042822

[36] Wen P.Y., , RANO 2.0: Update to the Response Assessment in Neuro-Oncology Criteria for High-and Low-Grade Gliomas in Adults. J Clin Oncol, 2023. 41(33): p. 5187–5199. 37774317 10.1200/JCO.23.01059 PMC10860967

[37] Fathi Kazerooni A., , Multiparametric MRI along with machine learning predicts prognosis and treatment response in pediatric low-grade glioma. Nature Communications, 2025. 16(1): p. 340. 10.1038/s41467-024-55659-z PMC11697432 39747214

[38] Davatzikos C., , AI-based prognostic imaging biomarkers for precision neuro-oncology: the ReSPOND consortium. Neuro Oncol, 2020. 22(6): p. 886–888. 32152622 10.1093/neuonc/noaa045 PMC7283022

[39] Sutherland I., Ulano A., and Thomas A.A., A volumetric analysis of timing and duration of T2/FLAIR changes on MRI following radiation therapy in patients with low-grade IDH-mutant glioma. Neurooncol Pract, 2025. 12(4): p. 631–636. 40814426 10.1093/nop/npaf024 PMC12349757

[40] Han H., , Clinical Validation of the Proenkephalin (PENK) Methylation Urine Test for Monitoring Recurrence of Non-muscle-invasive Bladder Cancer. Eur Urol Open Sci, 2024. 62: p. 99–106. 38496823 10.1016/j.euros.2024.02.010 PMC10940910

[41] Zhang H.P., , PENK inhibits osteosarcoma cell migration by activating the PI3K/Akt signaling pathway. J Orthop Surg Res, 2020. 15(1): p. 162. 32334633 10.1186/s13018-020-01679-6 PMC7183709

[42] Hildebrand B., , Expression of the proenkephalin gene in cultured astroglial cells: analysis of cell cycle dependence. Brain Res, 1997. 759(2): p. 285–91. 9221949 10.1016/s0006-8993(97)00268-0

[43] Kiyozuka K., , Apolipoprotein M supports S1P production and conservation and mediates prolonged Akt activation via S1PR1 and S1PR3. J Biochem, 2023. 174(3): p. 253–266. 37098187 10.1093/jb/mvad037

[44] Mahajan-Thakur S., , Sphingosine 1-phosphate (S1P) signaling in glioblastoma multiforme-A systematic review. Int J Mol Sci, 2017. 18(11). 10.3390/ijms18112448 PMC5713415 29149079

[45] Bai Y., , Magnetic resonance imaging to detect tumor hypoxia in brain malignant disease: A systematic review of validation studies. Clinical and Translational Radiation Oncology, 2025. 52: p. 100940. 10.1016/j.ctro.2025.100940 PMC11908384 40093743

[46] Liberti M.V. and Locasale J.W., The Warburg Effect: How Does it Benet Cancer Cells? Trends Biochem Sci, 2016. 41(3): p. 211–218. 26778478 10.1016/j.tibs.2015.12.001 PMC4783224

[47] Nakhle J., , Mitochondria Transfer from Mesenchymal Stem Cells Confers Chemoresistance to Glioblastoma Stem Cells through Metabolic Rewiring. Cancer Res Commun, 2023. 3(6): p. 1041–1056. 37377608 10.1158/2767-9764.CRC-23-0144 PMC10266428

[48] Watson D.C., , GAP43-dependent mitochondria transfer from astrocytes enhances glioblastoma tumorigenicity. Nat Cancer, 2023. 4(5): p. 648–664. 37169842 10.1038/s43018-023-00556-5 PMC10212766

[49] Cortes Ballen A.I., , Metabolic Reprogramming in Glioblastoma Multiforme: A Review of Pathways and Therapeutic Targets. Cells, 2024. 13(18). 10.3390/cells13181574 PMC11430559 39329757

[50] Löding S., , Blood based metabolic markers of glioma from pre-diagnosis to surgery. Scientific Reports, 2024. 14(1): p. 20680. 39237693 10.1038/s41598-024-71375-6 PMC11377417

[51] Xue C., , Mitochondrial pyruvate carrier 1: a novel prognostic biomarker that predicts favourable patient survival in cancer. Cancer Cell Int, 2021. 21(1): p. 288. 34059057 10.1186/s12935-021-01996-8 PMC8166087

[52] Chai Y., , MPC1 deletion is associated with poor prognosis and temozolomide resistance in glioblastoma. J Neurooncol, 2019. 144(2): p. 293–301. 31236818 10.1007/s11060-019-03226-8

[53] Karsy M., Guan J., and Huang L.E., Prognostic role of mitochondrial pyruvate carrier in isocitrate dehydrogenase-mutant glioma. J Neurosurg, 2019. 130(1): p. 56–66. 29547090 10.3171/2017.9.JNS172036

[54] Rong Y., , Analysis of the potential biological value of pyruvate dehydrogenase E1 subunit β in human cancer. World J Gastrointest Oncol, 2024. 16(1): p. 144–181. 38292838 10.4251/wjgo.v16.i1.144 PMC10824119

[55] Zhou H., , A comprehensive and systematic analysis of Dihydrolipoamide S-acetyltransferase (DLAT) as a novel prognostic biomarker in pan-cancer and glioma. Oncol Res, 2024. 32(12): p. 1903–1919. 39574473 10.32604/or.2024.048138 PMC11576973

[56] May J.L., , IDH3α regulates one-carbon metabolism in glioblastoma. Sci Adv, 2019. 5(1): p. eaat0456. 10.1126/sciadv.aat0456 PMC6314828 30613765

[57] Nagashima H., , Diagnostic value of glutamate with 2-hydroxyglutarate in magnetic resonance spectroscopy for IDH1 mutant glioma. Neuro Oncol, 2016. 18(11): p. 1559–1568. 27154922 10.1093/neuonc/now090 PMC5063515

[58] Scott A.J., , Metabolomic Proles of Human Glioma Inform Patient Survival. Antioxid Redox Signal, 2023. 39(13–15): p. 942–956. 36852494 10.1089/ars.2022.0085 PMC10655010

